# Development and Validation of a HPLC-UV Method for Extraction Optimization and Biological Evaluation of Hot-Water and Ethanolic Extracts of *Dendropanax morbifera* Leaves

**DOI:** 10.3390/molecules23030650

**Published:** 2018-03-13

**Authors:** Hyung-Jae Choi, Dae-Hun Park, Seung-Hui Song, In-Soo Yoon, Seung-Sik Cho

**Affiliations:** 1 Department of Pharmacy, College of Pharmacy and Natural Medicine Research Institute, Mokpo National University, Muan, Jeonnam 58554, Korea; qweert15@naver.com (H.-J.C.); tmdgml7898@naver.com (S.-H.S.); 2 Department of Nursing, Dongshin University, Naju, Jeonnam 58245, Korea; dhj1221@hanmail.net; 3 Department of Manufacturing Pharmacy, College of Pharmacy, Pusan National University, Geumjeong, Busan 46241, Korea

**Keywords:** *Dendropanax morbifera*, extraction optimization, HPLC, xanthine oxidase

## Abstract

*Dendropanax morbifera* Leveille (Araliaceae) has been used in traditional oriental remedies for cancer, inflammation, diabetes, and thrombosis. However, a validated analytical method, standardization, and optimization of extraction conditions with respect to biological activity have not been reported. In this study, a simple and validated HPLC method for identifying and quantifying active substances in *D. morbifera* was developed. Hot water and ethanolic *D. morbifera* leaf extracts from different production regions were prepared and evaluated with regard to their chemical compositions and biological activities. The contents of active compounds such as rutin and chlorogenic acid were determined in four samples collected from different regions. The 80% ethanolic extract showed the best antioxidant activity, phenolic content, reducing power, and xanthine oxidase (XO) inhibitory activity. The validated HPLC method confirmed the presence of chlorogenic acid and rutin in *D. morbifera* leaf extracts. The antioxidant and XO inhibitory activity of *D. morbifera* extract could be attributed to the marker compounds. Collectively, these results suggest that *D. morbifera* leaves could be beneficial for the treatment or prevention of hyperuricemia-related disease, and the validated HPLC method could be a useful tool for the quality control of food or drug formulations containing *D. morbifera*.

## 1. Introduction

*Dendropanax morbifera* Leveille is a subtropical evergreen tree which belongs to the family Araliacea, and it has been used in traditional medicines for the treatment of infectious diseases, dermatopathy, and headaches [1]. Previous studies have reported that *D. morbifera* exhibit various pharmacological effects, including antioxidant [2], antidiabetic [3], hepatoprotective [4], anticomplementary [5], and antiatherogenic activities [6]. *D. morbifera* leaf has also been traditionally used as a botanical remedy in Asia [7]. The efficacy of extracts and active constituents prepared with *D. morbifera* leaves as a medicinal source has been investigated in a few studies to date. Several research studies have reported various pharmacological activities of *D. morbifera* leaves and their active ingredients identified, which are listed in Table 1. Recently there has been a great effort to find candidates from natural products to effectively control metabolic disease [8].

*D. morbifera* leaves were reported to possess antioxidant and anticancer activities by moduating cellular apoptosis in various human tumor cell lines such as colon adenocarcinoma cells, biliary tract cells, hepatocellular carcinoma cells, and human osteocarcinoma cells [1]. Additionally, anti-inflammatory, antithrombotic, and other activities were found in *D. morbifera* leaf extracts, as shown in Table 1. However, the effects of *D. morbifera* leaf extracts on the activity of xanthine oxidase (XO) have not been reported. In the previous reports listed in Table 1, *D. morbifera* leaf extracts were prepared with organic solvents such as ethanol, methanol, and chloroform. However, there have been few studies on the optimization of extraction conditions with respect to biological activity, phytochemical contents, or both.

Recently, considerable effort has been focused on developing *D. morbifera* leaf as a therapeutic or functional source, but no positive results have been reported. To facilitate the pharmaceutical industrialization of *D. morbifera* leaf, a standardization process of the plant material with marker compounds identified using validated analytical methods is highly required. However, no validated analytical methods for the standardization and optimization of the biological properties of *D. morbifera* preparations have been reported in the literature. Here, we report the preparation of various hot water and ethanolic extracts of *D. morbifera* leaves collected from four different regions in Korea as well as the development of a simple and validated high-performance liquid chromatography (HPLC) method for chemical profiling and standardization of the plant extracts. In addition, the extraction process was optimized for phenolic contents and biological activities such as antioxidant and XO inhibitory activity.

## 2. Results and Discussion

### 2.1. Optimization of Chromatographic Conditions 

**Figure 1:**
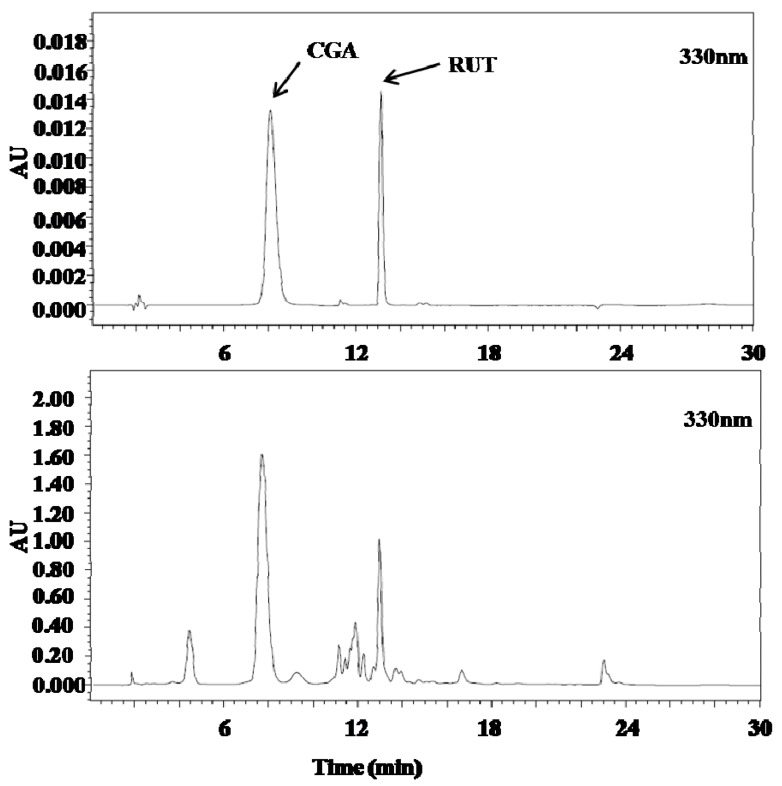
Chromatogram of standard and *D. morbifera* leaf extract.

The HPLC conditions were optimized for the mobile phase composition, column temperature, wavelength, and flow rate (data not shown). A gradient program was used to separate the active markers in a single run within a reasonable period. Detection wavelengths were set according to the ultraviolet (UV) absorption maxima of the compounds (330 nm). The chemical structures of the active constituents identified and representative chromatograms of the standard mixture and sample extracts are shown in Figure 1.

### 2.2. Method Validation

The limit of detection (LOD) of an analytical procedure is the lowest amount of an analyte in a sample that can be detected but not necessarily quantified [16] while the limit of quantification (LOQ) is the lowest level in the linear concentration range with acceptable precision and accuracy. The LOD of the present method was 0.65 and 0.50 μg/mL for chlorogenic acid and rutin, respectively (Table 2), and the corresponding LOQ values were 2.13 and 1.64 μg/mL, respectively (Table 2). Calibration curves were linear over a concentration range of 6.25–50 μg/mL for the two markers. The calibration curves exhibited good linear regressions (coefficient of determination *r*^2^ > 0.999 for chlorogenic acid and rutin, Table 2).

The results of the intraday and interday precision experiments are shown in Table 3. The developed method was precise, as indicated by the relative standard deviation (RSD) values (less than 2.5%) for the repeatability of the intraday and interday precision studies, which were below the limit recommended by the International Conference on Harmonisation (ICH) guidelines [17]. The overall recovery percentages were in the range of 92.36–98.12% for chlorogenic acid and 98.36–106.83% for rutin. These results demonstrate that the developed method was reproducible with good accuracy (Table 3).

The results of the repeatability experiments are shown in Table 4. The developed method was precise; the RSD values for the repeatability precision studies were below 2.0%.

### 2.3. Contents of Marker Compounds in D. morbifera from Different Cultivation Regions

**Figure 2:**
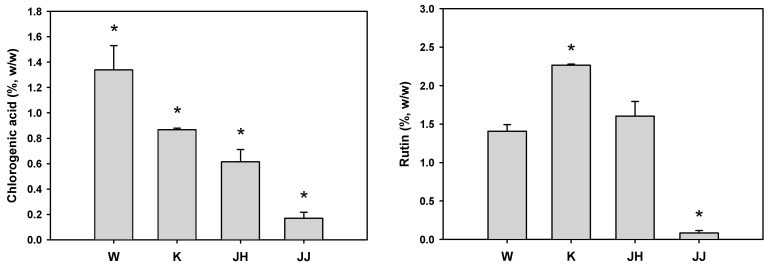
Contents of chlorogenic acid and rutin in hot water extracts of *D. morbifera* leaf from different cultivation regions. W: Wando, K: Kangjin, JH: Jangheung, JJ: Jeju. The asterisk represents a value significantly different from the other groups (*p* < 0.05). Values are mean ± standard deviation (*n* = 3).

Plant samples were collected from four different cultivation regions to compare the extraction yield and productivity of the active substances. The extraction yield and content of markers were compared for the various hot water extracts of the plant. The validated HPLC method was used to analyze the four different extracts. Chlorogenic acid and rutin were commonly identified in all samples. The extraction yield of samples from the various regions decreased in the following order: Jangheung (JH, 13.46%) > Wando (W, 12.03%) > Kangjin (K, 11.0%) > Jeju (JJ, 8.2%), indicating that the JJ sample showed the lowest yield. The average amounts (wt %) of chlorogenic acid and rutin are presented in Figure 2. 

The contents of chlorogenic acid were highest in the W sample, while the contents of rutin were highest in the K sample. The sum of the two active ingredient contents (chlorogenic acid and rutin) of W and K was comparable to each other, whereas the extraction yield of W was higher than that of K. Thus, *D. morbifera* from region W was used for further experiments.

### 2.4. Contents of Marker Compounds in D. morbifera Leaf Extracts

**Figure 3:**
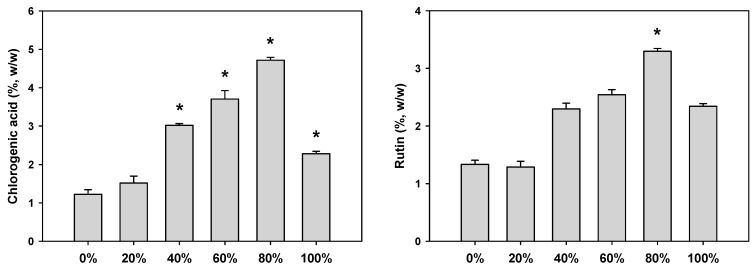
Contents of chlorogenic acid and rutin in hot water (0%) and ethanolic extracts (20–100%) of *D. morbifera* leaf. Ethanolic extracts were prepared with 20–100% ethanol. The asterisk represents a value significantly different from the other groups (*p* < 0.05). Values were the mean ± standard deviation (*n* = 3).

Six extracts, hot water and ethanolic extract with varying ethanol contents (20 to 100%, *v*/*v*) were prepared and compared with respect to the marker contets using the validated HPLC method. The average contents (wt %) of both markers are presented in Figure 3, and their levels in the 80% ethanolic extract were greater than those in the other ethanolic extracts (chlorogenic acid: 4.71 ± 0.06%; rutin: 3.29 ± 0.04%). Thus, 80% ethanol was selected as the most efficient extraction solvent.

### 2.5. Antioxidant Activity and Total Phenolic Contents of D. morbifera Extracts 

The antioxidant potential of various ethanoilc extracts of *D. morbifera* was determined using the 2,2-diphenyl-1-picrylhydrazyl (DPPH) scavenging and reducing power assays. The DPPH scavenging assay is a fast and easy method for evaluating the free radical scavenging ability of given samples. The antioxidant effects of plant extracts are generally related to the phenolic contents, and phenolic-rich sources of phytochemicals with antioxidant activity have curative benefits against conditions such as inflammation, oxidative stress, and other metabolic diseases [17].

**Figure 4:**
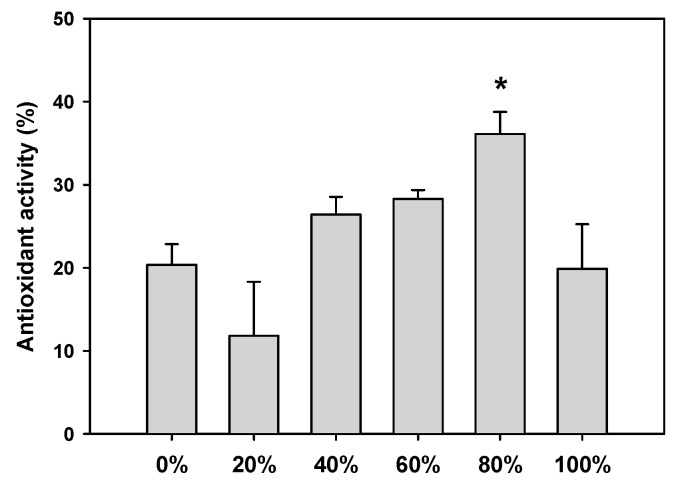
DPPH scavenging activity of ethanolic extracts of *D. morbifera* leaf. Ethanolic extracts were prepared with 20–100% ethanol. The asterisk represents a value significantly different from the other groups (*p* < 0.05). Values were the mean ± standard deviation (*n* = 3).

The antioxidative properties of the test plant extracts were closely correlated with the composition of active compounds such as phenolics. Therefore, we compared the phenolic contents (mg/g as gallic acid) of the various *D. morbifera* leaf extracts. The DPPH radical scavenging activity is shown in Figure 4. The 80% ethanolic extract showed the highest DPPH radical scavenging activity (36.10 ± 2.68%) with a half-maximal inhibitory concentration (IC_50_) of 116.97 μg/mL.

The reducing power assay is also very useful for evaluating the antioxidant activity. In the present study, we tested the reductive capability of extract sample by measuring the reduction of Fe^3+^. The 80% ethanolic extract exhibited the highest activity among the six extracts (Table 5). The reductive activity expressed as vitamin C equivalents was 17.70 ± 0.40 μg/100 μg. The total phenolic content was determined using the Folin–Ciocalteu method [17], and it was reported as gallic acid equivalents by referencing the standard curve (*r*^2^ > 0.999), as shown in Table 5. The phenolic content of the 80% ethanolic extract was higher than that of the other ethanolic extracts (57.89 ± 2.6 mg/g as gallic acid equivalents). Taken together, the results indicate that the DPPH radical scavenging activity, reducing power, and phenolic content were significantly higher in the 80% ethanolic extract than in the other extracts.

### 2.6. XO Inhibitory Activity of D. morbifera Extracts

**Figure 5:**
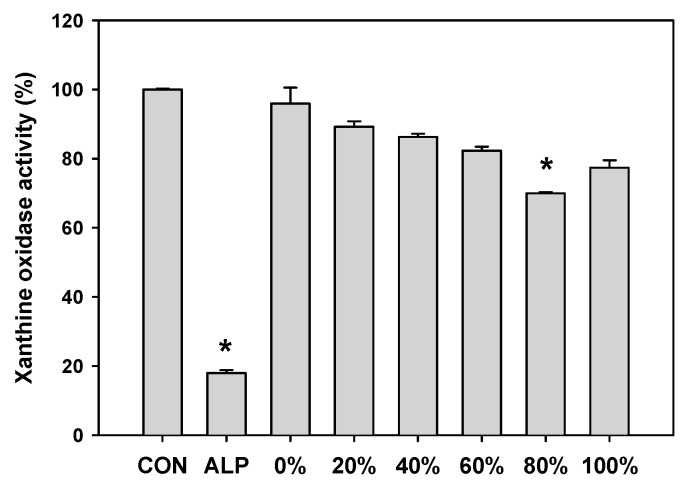
Xanthine oxidase inhibitory activities in ethanolic (0–100%) extracts of *D. morbifera* leaves (2 mg/mL) and allopurinol (ALP, 50 μg/mL). Ethanolic extracts were prepared with 20–100% ethanol. The asterisk represents a value significantly different from the other groups (*p* < 0.05). Values were the mean ± standard deviation (*n* = 3).

The effect of ethanol concentration on the XO inhibitory activity of *D. morbifera* leaf ethanolic extracts is shown in Figure 5. Allopurinol (ALP, positive control) at a concentration of 50 μg/mL significantly inhibited XO activity (82.08 ± 0.82%). The XO inhibitory activity of the ethanolic extracts was significantly higher in the 80% ethanolic extract than the other extracts. The XO inhibitory activity of the 80% ethanolic extract tended to increase in a concentration-dependent manner with increasing the extract concentration tested by up to 2 mg/mL (data not shown). Previously, we reported four different botanical extracts as potential XO inhibitors [18]. Yoon et al. [19,20] reported that the optimized extracts of *Corylopsis coreana* and *Camellia japonica* inhibited XO activity by approximately 50% at a concentration of 2 mg/mL. Additionally, Yoon et al. [18] demonstrated that *Quercus acuta* extract showed approximately 50% XO inhibitory activity at a concentration of 1 mg/mL. *Cudrania tricuspidata* extract inhibited XO by approximately 75% at a concentration of 2 mg/mL [21]. The plant extracts with XO inhibitory activity at 1 and 2 mg/mL demonstrated consistent effects in the in vivo animal disease model. Thus, it is plausible that the 80% ethanolic extract of *D. morbifera* leaves could be developed as a candidate antihyperuricemic agent.

We identified chlorogenic acid and rutin as marker compounds of extracts of *D. morbifera* leaves from different cultivation regions. In the present study, we confirmed that these two compounds were common constituents of the *D. morbifera* leaves from all four regions. This finding could be important in the use of this plant for industrial purpose. Furthermore, we established the optimal analysis methods and optimized the extraction conditions for further studies. Rutin is a flavonoid known to have diverse pharmacological effects such as antihyperuricemia, anti-inflammatory, anticonvulsant, and anti-Alzheimer’s disease [19,22,23,24]. A previous study reported that rutin at doses of 50 and 100 mg/kg markedly reduced biological markers in hyperuricemic mice [23]. In another study, rutin exhibited antihyperuricemic effects by inhibiting xanthine dehydrogenase/XO activity [24]. Chlorogenic acid has also been reported to have diverse pharmacological effects such as anti-inflammatory, antiallergic, and antihyperuricemic effects [25,26,27,28]. Meng et al. [27] reported that chlorogenic acid has anti-gout activity by inhibiting XO activity. Chlorogenic acid also decreased the levels of proinflammatory cytokines such as interleukin (IL)-1β, IL-6, and tumor necrosis factor (TNF)-α induced by uric acid [27,28,29]. These results suggest that chlorogenic acid may have considerable potential for development as an antihyperuricemic agent.

As shown in Table 1, previous studies reported the diverse activities of extracts of *D. morbifera* leaves. However, to the best of our knowledge, our present study is the first to report the optimization of the extraction process of pharmaceutically active indicators from *D. morbifera* leaves from various regions and the comparation of antioxidant and XO inhibitory activities of various region-specific extracts.

A standard analytical method is crucial for the industrial application of plant extracts, and extraction optimization is an essential process for the optimization and quality control of natural products from various sources. However, there has been no report of a standard profile for *D. morbifera*. Moreover, currently, no other validated HPLC method has been reported for the simultaneous determination of chlorogenic acid and rutin in the *Dendropanax* genus. In Korea, one species of *D. morbifera* is mainly cultivated in four different regions studied (W, K, JH, and JJ). In this study, the differences in the marker content of samples from the four production regions were compared using our validated HPLC methods, which showed efficiency in the analysis and optimization of the *D. morbifera* leaf preparations. Moreover, the findings of the present study could be applied to the industrialization of *D. morbifera* by providing basic information on samples from the four cultivation regions in Korea.

## 3. Experimental Section

### 3.1. Plant Material and Extract Preparation

*D. morbifera* leaves were collected in October 2016 near Wando (34.3110596 N, 126.755054 E), Kangjin (34.642077 N, 126.76726 E), and Jangheung (34.681685 N, 126.906927 E) in Jeonnam Province, Korea and Jeju (33.499621 N, 126.531188 E) in Jeju Province, Korea. Voucher specimens (MNUCSS-DMWD-01, MNUCSS-DMJH-01, MNUCSS-DMHN-01, and MNUCSS-DMJJ-01) were deposited at the College of Pharmacy, Mokpo National University (Muan, Korea). For the present study, the air-dried, powdered *D. morbifera* leaves (50 g) were extracted twice with 20–100% ethanol (300 mL) at room temperature for 3 days. The 0% extract was prepared as hot-water extract (100 °C, 4 h). After filtration, the resultant ethanol solution was evaporated, freeze-dried, and stored at −50 °C. The crude extract was resuspended in ethanol and filtered using a 0.4-μm membrane. All the samples were subjected to extraction optimization and used in the in vitro experiments.

### 3.2. Chromatographic Conditions 

All analyses were performed using an Alliance 2695 HPLC system (Waters, Milford, MA, USA) equipped with a photodiode array detector. A revese phase C18 analytical column (5-μm, 150 mm × 5 mm) was used with a mobile phase consisting of a mixture of solvent A (acetonitrile) and B (0.2% phosphoric acid). A gradient elution (from 10/90 to 80/20 *v/v*) at a flow rate of 0.8 mL/min was used and the analytical conditions are described in Table 6. 

### 3.3. Method Validation

The analytical method used for the quantification of chlorogenic acid and rutin in the *D. morbifera* leaf extracts was validated for specificity, linearity, sensitivity, accuracy, precision, and recovery, as previously described [17].

### 3.4. Analysis of D. morbifera Leaf Extracts

The HPLC method developed inthis study was used to quantitatively determine the chlorogenic acid and rutin contents in the extracts of *D. morbifera* leaves.

### 3.5. DPPH Free Radical Assay 

The DPPH radical scavenging assay was used to evaluate the antioxidant properties of the extracts. Briefly, various concentrations of the ethanolic extract solutions (0.5 mL) were mixed with 0.4 mM DPPH (0.5 mL) for 10 min. The absorbance at 517 nm was measured using a microplate reader (Perkin Elmer, Waltham, MA, USA). The radical scavenging activity was calculated as a percentage using the following equation:

DPPH radical scavenging activity (%) = [1 − (A_sample_/A_blank_)] × 100 (1)

IC_50_ (μg/mL) values were calculated from the data of the DPPH free radical scavenging activities of the various samples [17].

### 3.6. Reducing Power

The reducing power assay was also used to evaluate the antioxidant properties of the extracts. The extract (0.1 mL) was mixed with 0.2 M sodium phosphate buffer (0.5 mL), and 1% potassium ferricyanide (0.5 mL), followed by incubation at 50 °C for 20 min and the reaction was stopped by adding 10% trichloroacetic acid solution (0.5 mL). The mixture was centrifuged at 2000× *g* for 10 min, the supernatant was mixed with distilled water (0.5 mL) and 0.1% iron (III) chloride solution (0.1 mL), and the absorbance of the final mixture was measured at 700 nm. The reducing powers of the various samples were expressed as vitamin C equivalents [17].

### 3.7. Total Phenolic Content

The total phenolic content was determined using the Folin-Ciocalteu assay. An extract solution (1 mL) or standard (gallic acid) was mixed with 1 mL each of 2% sodium carbonate and 10% Folin-Ciocalteu phenol reagent for 10 min. The absorbance of the mixture was then measured at 750 nm using a microplate reader (Perkin Elmer, Waltham, MA, USA). The measurement was compared to a calibration curve constructed using gallic acid standard concentrations. The results were expressed as milligrams of gallic acid equivalents per gram of the sample [17].

### 3.8. XO Inhibitory Activity In Vitro

The XO inhibitory activity was measured according to a previous report [19]. Briefly, 0.1 mL of each sample was mixed with 0.6 mL phosphate buffer (100 mM, pH 7.4), 0.1 mL XO (0.2 U/mL), and 0.2 mL xanthine (1 mM, dissolved in 0.1 M sodium hydroxide [NaOH]) for 15 min. The reaction was stopped by adding 0.2 mL 1 M hydrochloric acid (HCl), and the absorbance was measured at 290 nm with ALP as the positive control.

### 3.9. Statistical Analysis

All data were expressed as mean ± standard deviation, and analysis of variance (post-hoc Tukey’s multiple range test) was performed using the Statistical Package for the Social Sciences software (version 12.0, IBM Co., Armonk, NY, USA). A p-value less than 0.05 was considered to be statistically significant.

## 4. Conclusions

In the present study, hot water and ethanolic extracts of *D. morbifera* leaf extracts from four different cultivation regions were successfully prepared, and their chemical profiles and biological activities were evaluated. Hot water extracts from JH showed the highest yield and extract of the W and K samples showed high concentrations of the selected makers. The 80% ethanolic extract exhibited the most potent DPPH radical scavenging activity, reducing power, phenolic content, and XO inhibitory activity. The developed HPLC method was validated and applied to identify chlorogenic acid and rutin, which were found to be common constituents of all the *D. morbifera* leaf extracts. These findings suggest that the observed antioxidant and XO inhibitory activities of *D. morbifera* extracts were attributed, at least in part, to the marker compounds. To the best of our knowledge, this is the first study to report on a validated analytical method for the standardization and optimization of the biological properties of *D. morbifera* preparations. Further investigation is warranted to confirm the in vivo pharmacological activity of *D. morbifera* extract and its two constituents and assess the safe use of the plant. These propose efforts could lead to the development of *D. morbifera* as a potential, effective antioxidant and anti-hyperuricemic/gout agent.

## Figures and Tables

**Table 1:** Pharmacological activities and/or chemical constituents of *D. morbifera* leaf extracts reported in previous literatures.

Ext. Solvent	Activity	Chemical Identified	Region	Ref.
chloroform	anti-inflammatory	-	-	[4]
70% ethanol	anticancer	-	-	[9]
-	anti-inflammatory	oleifoliside A	-	[10]
chloroform	kidney damage	-	-	[11]
-	anticomplement	three polyacetylenes	-	[5]
methanol	anti-inflammatory	rutin and 21 compounds	Jeju, Korea	[12]
80% ethanol	antioxidant anticancer	chlorogenic acid caffeic acid, rutin, rosmarinic acid	Jeju, Korea	[1]
-	Antithrombotic	rutin	Wando, Korea	[13]
-	Anticancer	oleifoliside B	-	[14]
-	Neurogenerative	rutin	Wando, Korea	[15]

**Table 2:** HPLC data of calibration graphs and limit of quantification and detection of two markers.

Analyte	Retention Time (min)	*r*^2^	Range (μg/mL)	LOQ (μg/mL)	LOD (μg/mL)
Chlorogenic acid	7.7	0.9988	3.125–50	2.13	0.65
Rutin	12.7	0.9996	3.125–50	1.64	0.50

**Table 3:** Analytical results of intra-day and inter-day precision and accuracy.

Analyte	Conc. (μg/mL)	Intra-Day (*n* = 3)		Inter-Day (*n* = 3)	
		RSD (%)	Accuracy (%)	RSD (%)	Accuracy (%)
Chlorogenic acid	12.5	4.73	92.36	1.17	94.78
	25	1.11	95.70	1.64	97.07
	50	2.12	96.12	1.95	98.12
Rutin	12.5	3.18	100.65	7.15	106.83
	25	1.00	99.07	5.71	102.17
	50	1.19	98.36	1.83	100.60

**Table 4:** Analytical recovery data (*n* = 6).

Analyte	Added (μg/mL)	Recovery (%) (Mean ± SD)	RSD (%)
Chlorogenic acid	12.5	95.77 ± 0.62	0.78
	25	96.72 ± 1.33	1.50
	50	98.21 ± 0.45	0.48
Rutin	12.5	102.73 ± 0.40	0.40
	25	97.65 ± 1.49	1.56
	50	98.64 ± 0.43	0.43

**Table 5:** Reducing power and total phenolic contents of *D. morbifera* leaf extracts.

Extract	Reducing Power (Ascorbic Acid eq. μg/100 μg Extract)	Total Phenolic Content (Gallic Acid eq. mg/g)
Water	8.4 ± 0.2	30.27 ± 0.6
20% EtOH Ex	7.4 ± 0.7	26.3 ± 0.6
40% EtOH Ex	12.3 ± 0.5	39.28 ± 1.4
60% EtOH Ex	13.6 ± 0.2	52.30 ± 2.9
80% EtOH Ex	17.7 ± 0.4	57.89 ± 2.6
100% EtOH Ex	5.5 ± 1.3	34.72 ± 1.3

**Table 6:** Analytical conditions of HPLC system for analyzing two markers.

Parameters	Conditions		
Column	Zorbax extended-C18 (C18, 4.6 mm × 150 mm, 5 µm)		
Flow rate	0.8 mL/min		
Injection volumn	10 μL		
UV detection	330 nm		
Run time	30 min		
Gradient	**Time (min)**	**% A**	**% B**
	0	10	90
	7	10	90
	8	20	80
	20	25	75
	21	100	0
	25	10	90
	30	10	90
